# New improved radiometabolite analysis method for [^18^F]FTHA from human plasma: a test-retest study with postprandial and fasting state

**DOI:** 10.1186/s13550-024-01114-5

**Published:** 2024-06-13

**Authors:** Richard Aarnio, Anna Kirjavainen, Johan Rajander, Sarita Forsback, Kari Kalliokoski, Pirjo Nuutila, Zvonko Milicevic, Tamer Coskun, Axel Haupt, Iina Laitinen, Merja Haaparanta-Solin

**Affiliations:** 1 MediCity Research Laboratory, University of Turku, Turku, Finland; 2 Drug Research Doctoral Programme, University of Turku, Turku, Finland; 3 Turku PET Centre, University of Turku, Kiinamyllynkatu 4-8, Turku, FI-20520 Finland; 4 Accelerator Laboratory, Turku PET Centre, Åbo Akademi University, Turku, Finland; 5 Department of Endocrinology, Turku University Hospital, Turku, Finland; 6 Eli Lilly and Company, Indianapolis, IN USA; 7 Antaros Medical AB, Bioventure Hub, Mölndal, Sweden

**Keywords:** [^18^F]FTHA, Radiometabolite analysis, Parent fraction, Radio-TLC, Metabolic imaging

## Abstract

**Background:**

Fatty acid uptake can be measured using PET and 14-(*R,S*)‐[^18^F]fluoro‐6‐thia‐heptadecanoic acid ([^18^F]FTHA). However, the relatively rapid rate of [^18^F]FTHA metabolism significantly affects kinetic modeling of tissue uptake. Thus, there is a need for accurate chromatographic methods to analyze the unmetabolized [^18^F]FTHA (parent fraction). Here we present a new radiometabolite analysis (RMA) method, with comparison to a previous method for parent fraction analysis, and its use in a test-retest clinical study under fasting and postprandial conditions. We developed a new thin-layer chromatography (TLC) RMA method for analysis of [^18^F]FTHA parent fraction and its radiometabolites from plasma, by testing stationary phases and eluent combinations. Next, we analyzed [^18^F]FTHA, its radiometabolites, and plasma radioactivity from subjects participating in a clinical study. A total of 17 obese or overweight participants were dosed with [^18^F]FTHA twice under fasting, and twice under postprandial conditions and plasma samples were obtained between 14 min (mean of first sample) and 72 min (mean of last sample) post-injection. Aliquots of 70 plasma samples were analyzed using both methods, enabling head-to-head comparisons. We performed test-retest and group comparisons of the parent fraction and plasma radioactivity.

**Results:**

The new TLC method separated seven [^18^F]FTHA radiometabolite peaks, while the previous method separated three. The new method revealed at least one radiometabolite that was not previously separable from [^18^F]FTHA. From the plasma samples, the mean parent fraction value was on average 7.2 percentage points lower with the new method, compared to the previous method. Repeated [^18^F]FTHA investigations on the same subject revealed reproducible plasma SUV and parent fractions, with different kinetics between the fasted and postprandial conditions.

**Conclusions:**

The newly developed improved radio-TLC method for [^18^F]FTHA RMA enables accurate parent fraction correction, which is required to obtain quantitative data for modelling [^18^F]FTHA PET data. Our test-retest study of fasted and postprandial conditions showed robust reproducibility, and revealed clear differences in the [^18^F]FTHA metabolic rate under different study settings.

**Trial registration:**

EudraCT No: 2020-005211-48, 04Feb2021; and Clinical Trials registry NCT05132335, 29Oct2021, URL: https://classic.clinicaltrials.gov/ct2/show/NCT05132335.

**Supplementary Information:**

The online version contains supplementary material available at 10.1186/s13550-024-01114-5.

## Background

Free fatty acids (FFAs) are aliphatic chains that include a carboxylic acid group. Also known as non-esterified fatty acids (NEFAs), FFAs are building blocks for lipid components, together with glycerol. Lipolysis in adipose tissue releases FFAs into the blood [1]. FFAs are used for cellular energy through beta-oxidation in the mitochondria; for energy storage in various tissues; and as building blocks for hormones, proteins, and various lipids [2–4]. Radiolabeling FFAs with positron-emitting radionuclides enables positron emission tomography (PET) imaging of multiple processes occurring in a living organism by measuring the uptake of PET tracers, such as [^11^C]palmitate [5] and 14(*R,S*)-[^18^F]fluoro-6-thia-heptadecanoic acid ([^18^F]FTHA).

The first study using [^18^F]FTHA was published in 1991 [6] and since then, [^18^F]FTHA has been used to quantify FFA uptake in PET imaging studies, and can be produced under GMP conditions [7]. The latest advancement in [^18^F]FTHA radiosynthesis techniques has been to develop cassette-based systems [8]. When a cell uses [^18^F]FTHA for energy through the beta-oxidation reaction, the fluorine-18 is trapped in the mitochondria; thus, in myocardium, [^18^F]FTHA can be used to measure beta-oxidation in the mitochondria. On the other hand, in skeletal muscle, [^18^F]FTHA reveals a more general FFA uptake rather than only the uptake into mitochondria, as demonstrated with an intracellular [^18^F]FTHA radiometabolite analysis (RMA) method [9]. The total uptake into the cell also includes different FFA utilizations—for example, the formation and storage as triglycerides, glycerol esters, and phospholipids, as well as energy consumption [10]. PET imaging using [^18^F]FTHA as a surrogate for FA metabolism and/or uptake has been applied to study several organs, including white adipose tissue [11], brown adipose tissue [12], liver [13], brain [14], myocardium [4, 15], and skeletal muscle [16, 17].

FFA uptake in tissues is quantified as the measured fractional net influx rate (K_i_) of [^18^F]FTHA into organs, obtained from modelled imaging data using a Patlak model and plasma input, multiplied by the FFA level in the plasma. Notably, the rapid metabolism of [^18^F]FTHA has a significant impact on modeling. To quantify the true input function, the fraction of unmetabolized [^18^F]FTHA (parent fraction) in plasma must be analyzed over the time of interest, to perform metabolite-correction of the plasma time-activity curve (TAC) [13]. Several approaches to RMA have been described over the past decades, leading also to different methods of tracer uptake quantification [3, 15, 16, 18, 19]. The methods have primarily utilized reverse-phase C_18_-modified silica gel as the stationary phase; however, many publications lack key information required to replicate the sample preparation and RMA methods and their resulting radiochromatograms.

In the present study, we developed an improved RMA method to enable accurate data analysis from a [^18^F]FTHA PET study. A new method was needed because a change in the manufacturing process of the thin-layer chromatography (TLC) plate altered the chromatographic performance of the new plate lots, leading to insufficient resolution (R_s_) between [^18^F]FTHA and its radiometabolites, when using a previous TLC method [15, 18]. The new method enabled new separation possibilities, yielding improved separation of the radiometabolites. We further assessed the test-retest variability of RMA and plasma standardized uptake value (SUV, plasma radioactivity normalized according to injected radioactivity and body weight) in a [^18^F]FTHA clinical repeatability study. Here we present the new RMA method, with comparison to the previously used method, using thin-layer chromatography combined with digital autoradiography (radio-TLC) for parent fraction correction, along with a [^18^F]FTHA test-retest study under fasting and postprandial conditions.

## Methods

### Radiochemistry

[^18^F]FTHA was synthesized at the Radiopharmaceutical Chemistry Laboratory of Turku PET Centre (Turku, Finland), using an automated method under GMP conditions [7]. For this study, 42 synthesis batches were prepared, which showed mean radiochemical purity of 99.3 ± 0.2% (range, 98.8–99.7%).

### Study subjects

Parent [^18^F]FTHA, its radiometabolites, and plasma radioactivity were analyzed from 17 obese or overweight participants (BMI = 32.3 ± 3.4 kg/m^2^) during a clinical study (NCT05132335, EudraCT No: 2020-005211-48). All subjects gave their written consent, and the study plan was approved by the ethical committee of the Hospital District of South-Western Finland. The subjects underwent repeated dynamic [^18^F]FTHA PET (2.7 ± 0.2 MBq/kg) examinations—twice under fasting conditions (visits 1 and 2) and twice under postprandial conditions (visits 3 and 4), with the retest occurring around one week after the first examination. Postprandial assessment was performed approximately 110 min after a 400-kcal liquid meal (Resource 2.0, Nestlé Health Science, Vevey, Switzerland).

### Plasma samples

At approximately 14, 24, 31, 39, 46, 54, and 71 min post-injection, arterialized blood samples were collected from the warmed antecubital vein, using a catheter, into heparinized tubes (Vacutainer, Becton, Dickinson and Company, Franklin Lakes, NJ, USA). Blood samples were centrifuged (4°C, 2118 × *g*, 5 min). Aliquots of plasma were immediately used for RMA and for radioactivity measurement (Wizard 1480 3”; PerkinElmer, Turku, Finland), as described below.

### Radioactive standard

A [^18^F]FTHA sample was used as a radioactive standard, and was applied to each TLC plate to identify the retardation factor of the parent fraction. The standard was prepared in a plasma supernatant obtained from the subject prior to [^18^F]FTHA injection, such that it matched the matrix of the RMA samples.

### Plasma RMA

In both methods, protein was removed from the plasma samples by precipitation. A 250-µL aliquot of plasma was mixed with 350 µL of solvent (methanol/acetic acid in a ratio of 100:0.4, *v/v*), this mixture was vortexed and centrifuged (+ 22 °C, 14,100 × *g*, 90 s), and then the supernatant was separated. We compared the protection of [^18^F]FTHA from decomposition with and without the addition of non-radiolabeled FTHA (1:10000 *w*/*v*).

In the newly developed RMA method, the sample and a [^18^F]FTHA standard were each applied onto a HPTLC plate (silica gel 60 RP-18, art no. 1.05914.0001; Merck KGaA, Darmstadt, Germany; manufactured after December 2020). To concentrate the sample, we applied two 8-µL aliquots of the supernatant to the same spot on the plate, with a short drying period in between. The plate was dried, and then eluted with methanol/water/acetic acid (78:22:0.13, *v/v/v*) until the solvent reached 6 cm above the application line (travel time 35–40 min). Then the plate was dried, exposed to a recently erased autoradiography imaging plate (BAS-TR2025; Fuji Photo Film Co., Ltd., Tokyo, Japan), scanned with a BAS-5000 phosphorimager (Fuji Photo Film Co., Ltd., Tokyo, Japan), and analyzed using Aida Image Analyzer (v.4.22; Elysia-Raytest, GmbH, Straubenhardt, Germany). For each detectable peak, the fractions were calculated as the percentage of total ^18^F-radioactivity, at each individual sample time-point.

The previously used method was based on a published RMA method [15, 18], with modifications. We applied one 15-µL aliquot of the supernatant and the radioactive standard to the HPTLC plate (manufactured before December 2020). Then the plate was dried, and eluted with methanol/water/acetic acid (100:5:0.13, *v/v/v*) (4 cm, travel time 16–18 min). Finally, the plates were analyzed with digital autoradiography, as described above.

Using the previously used method with HPTLC plates manufactured after December 2020, after the manufacturing process was changed (communication from manufacturer), resulted in insufficient R_s_ between the [^18^F]FTHA and its radiometabolites. This discovery prompted our development of the new RMA method, which was optimized by testing new stationary phase and eluent combinations. We also tested the protein precipitation solvent composition and precipitation solvent-to-plasma ratio to achieve the highest radioactivity-to-sample volume ratio (methods described in Supplemental Table 1). The previously applied sample preparation was found to be the best option, and thus was used in the new method.

The previous RMA method was used for the analysis of 11 subjects (a total of 22 fasted and 22 postprandial visits). To facilitate comparison with the subsequent 6 subjects (a total of 12 fasted and 12 postprandial visits), for which the new RMA method was employed, we determined a correction factor. In 10 visits (a total of 5 fasted and 5 postprandial visits), both methods were concurrently employed for a comprehensive evaluation.

### Parent fraction and plasma SUV calculation

The percentage of the [^18^F]FTHA parent fraction over the total ^18^F-radioactivity in the sample was analyzed for each sample time-point, from each visit and each subject. These data were displayed as parent fraction curves. The area under the curve (AUC) was calculated for the parent fraction curves over a fixed period (0–60 min).

To perform head-to-head comparison of the parent fraction determined using the newly developed method versus the previous method, we ran 70 sample aliquots, taken at sequential time-points from 5 fasting and 5 postprandial visits, side-by-side with both methods. To enable between-group comparisons of the parent fraction among all subjects, we calculated a correction factor, which was used to enable converting parent fraction values from the previous method to the new method.

We calculated the plasma SUV and the corresponding AUC (over fixed time-points of 14–60 min) for all individual visits. Plasma SUV was corrected for decay, and for the remaining radioactivity in the injection syringe, cannula, and tubing (supplemental materials).

The results from subjects undergoing the same protocol were pooled for comparisons between the fasted and postprandial protocols. Repeatability was analyzed between the individual test and retest visits.

### Statistical analysis

All results are reported as mean ± standard deviation (SD). GraphPad Prism 9 (GraphPad Software, San Diego, CA) was used for statistical calculations and AUC integration. For paired comparisons student parametric t-test was used when *n* ≥ 30 and a non-parametric Wilcoxon matched-pairs signed rank test for smaller sample size tests. For unpaired group analysis parametric t-tests were used. Differences were considered significant when *p* < 0.05.

## Results

### Analysis of [^18^F]FTHA RMA methods

**Fig. 1:**
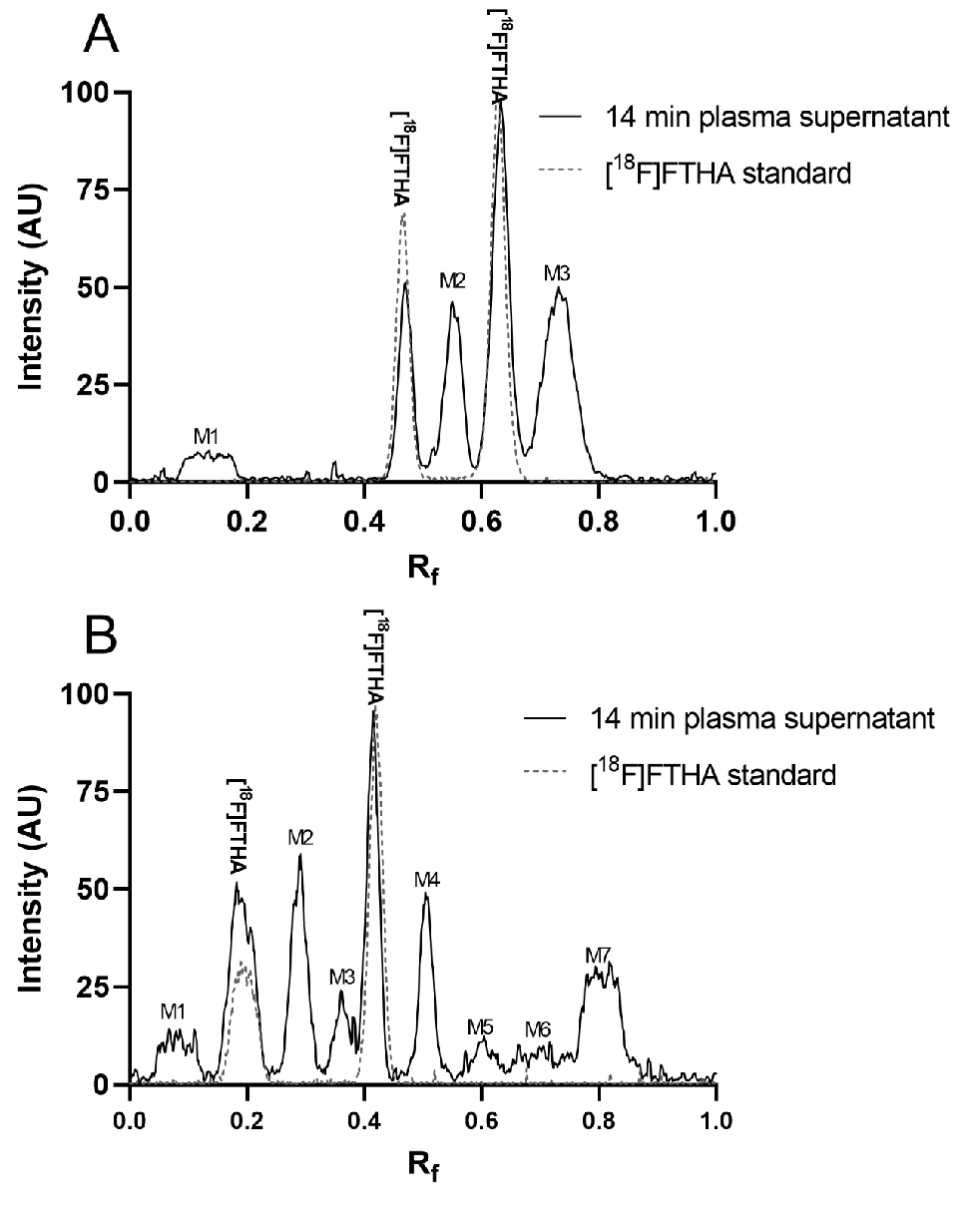
Representative radiochromatograms of the 14 min plasma supernatant (continuous line) and [^18^F]FTHA standard sample (dashed line) using the previous method (**A**) and new method (**B**). The intensities of the standard and plasma samples were normalized

The new method displayed up to nine peaks, whereas the previous method showed sufficient separation of only five peaks (Fig. 1 and Supplemental Fig. 1). In both methods, two of the peaks corresponded to [^18^F]FTHA, according to the [^18^F]FTHA standard. Notably, the radioactive [^18^F]FTHA standard decomposes into two separate bands on the TLC plate, possibly indicating formation of [^18^F]FTHA sulfoxide [20]. We found that [^18^F]FTHA decomposition decreased by adding non-radiolabeled FTHA as a carrier to the precipitation solvent, keeping samples at a cold temperature, and applying samples to the C_18_-modified HPTLC plate immediately before drying and eluting. When considering both bands indicated by the standard to be non-metabolized [^18^F]FTHA, the parent fraction sum remained unaffected (tested with duplicate plates under varying conditions, data not shown). With the new method, the fraction of radioactivity of both bands corresponding to [^18^F]FTHA in the metabolite samples showed a continuously decreasing trend over sample time-points, suggesting that neither band includes co-eluting radiometabolites.

### Parent fraction in plasma

**Fig. 2:**
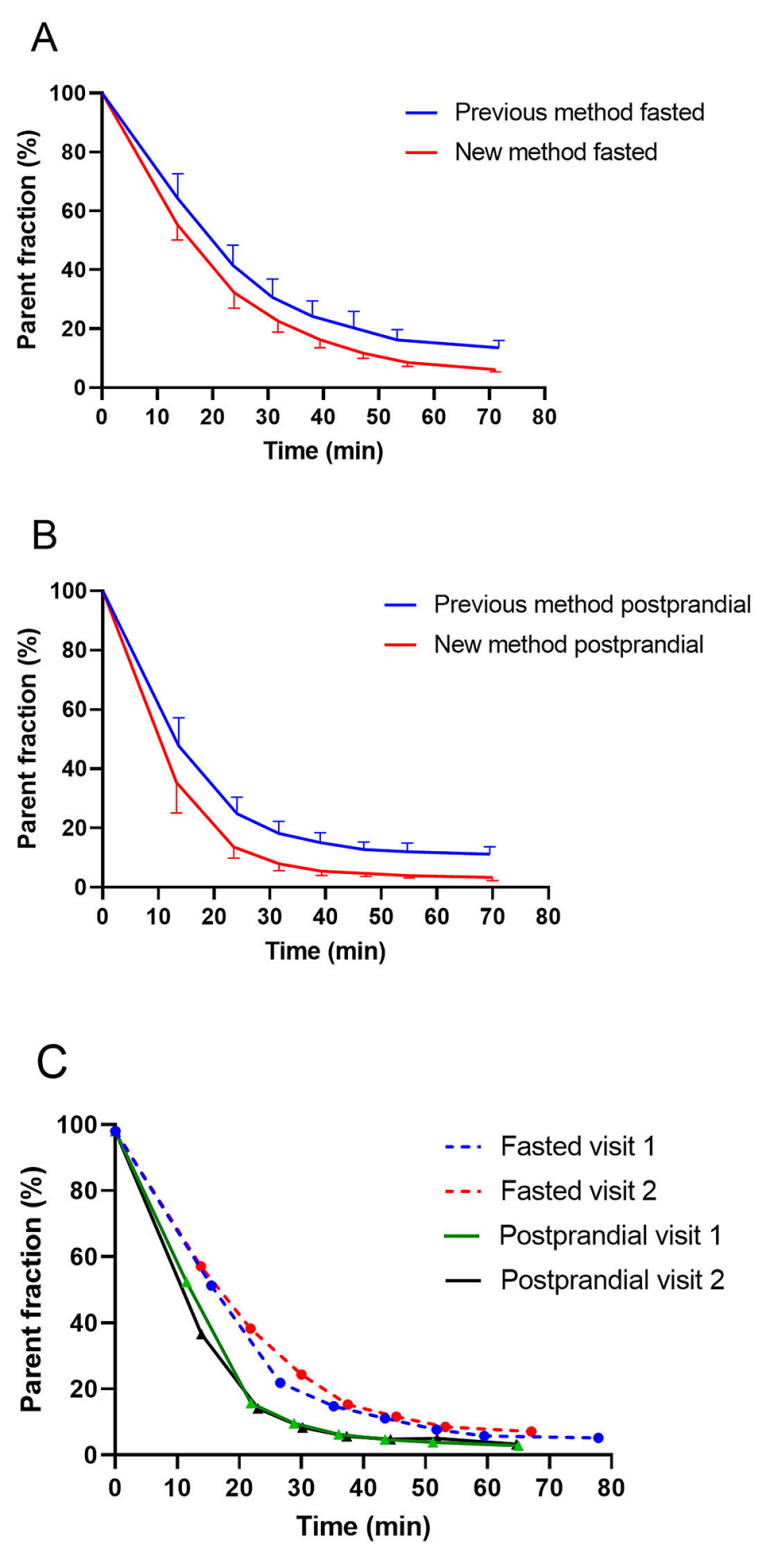
Percentages of unchanged [^18^F]FTHA out of all radioactivity in the sample. (**A**, **B**) Parent fraction (mean and SD) analyzed using the previous method (blue) and new method (red), during fasted visits (*n* = 25 fasting visits and previous method; *n* = 13 fasting visits and new method) (**A**) and postprandial visits (*n* = 26 postprandial visits and previous method; and *n* = 11 postprandial visits and new method) (**B**). The representative parent fraction curves were generated using the new method and from multiple visits of a single subject; twice during fasting, indicated by dashed blue and red lines, and twice during postprandial, represented by black and green lines (**C**)

The parent fraction in plasma samples over time (parent fraction curve) demonstrated the rapid metabolism of [^18^F]FTHA within 30 min. With the new method, the mean parent fraction values at 30 and 70 min, respectively, were 24.3 ± 3.6% and 6 ± 1.2% for fasted visits, and 8.5 ± 2.4% and 3.1 ± 1.0% for postprandial visits (Fig. 2). With the previous method, the mean parent fraction values at 30 and 70 min, respectively, were 31.0 ± 5.2% and 13.3 ± 2.2% for fasted visits, and 18.8 ± 4.3% and 11.1 ± 2.4% for postprandial visits (Fig. 2). Thus, the new method revealed that [^18^F]FTHA metabolism was faster than previously thought, based on the markedly lower parent fraction values.

**Fig. 3:**
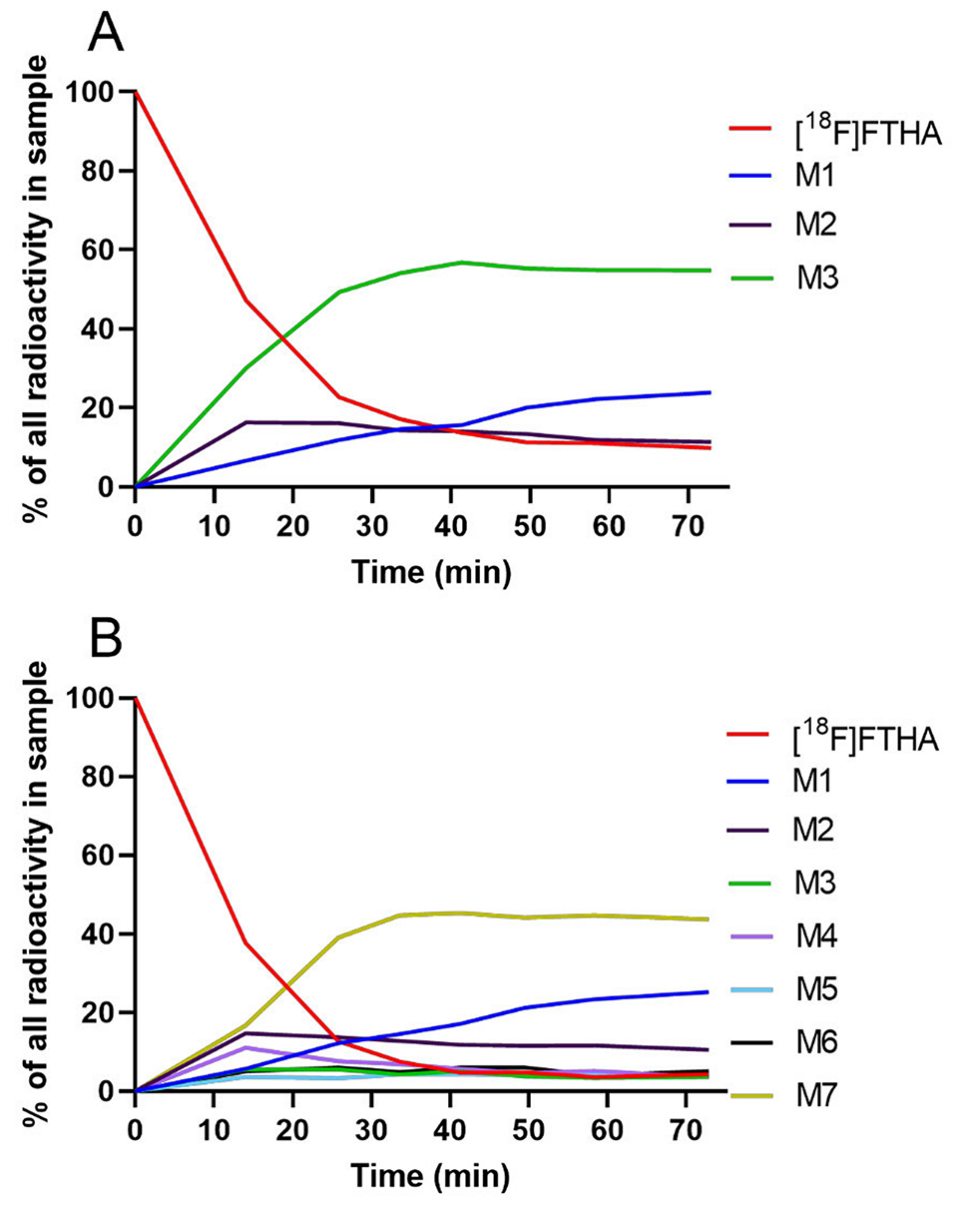
Representative percentage of radioactivity over time of [^18^F]FTHA and its radiometabolites (M1–M7) in plasma, from plasma samples obtained during postprandial imaging, using the previous method (**A**) and the new method (**B**)

Some of the newly separated peaks in the new method are only a result of radiometabolites separating from each other that travelled together on the TLC plate and were not separable from each other with the previous method. However, the radiometabolites separated using the new method included at least one new radiometabolite that was not separable from [^18^F]FTHA using the previous method (Fig. 3); therefore, the parent fraction curve also plateaued much closer to zero. To quantify the difference in the parent fraction result with the newly separable radiometabolite(s), we analyzed 70 plasma samples side-by-side using both methods. Compared to the new method, the previous method showed that the average AUCs were 16.2 ± 5.5% higher for fasted visits *(p* = 0.06, *n* = 5), and 37.2 ± 7.0% higher for postprandial visits (*p* = 0.06, *n* = 5) (Supplemental Table 2).

**Fig. 4:**
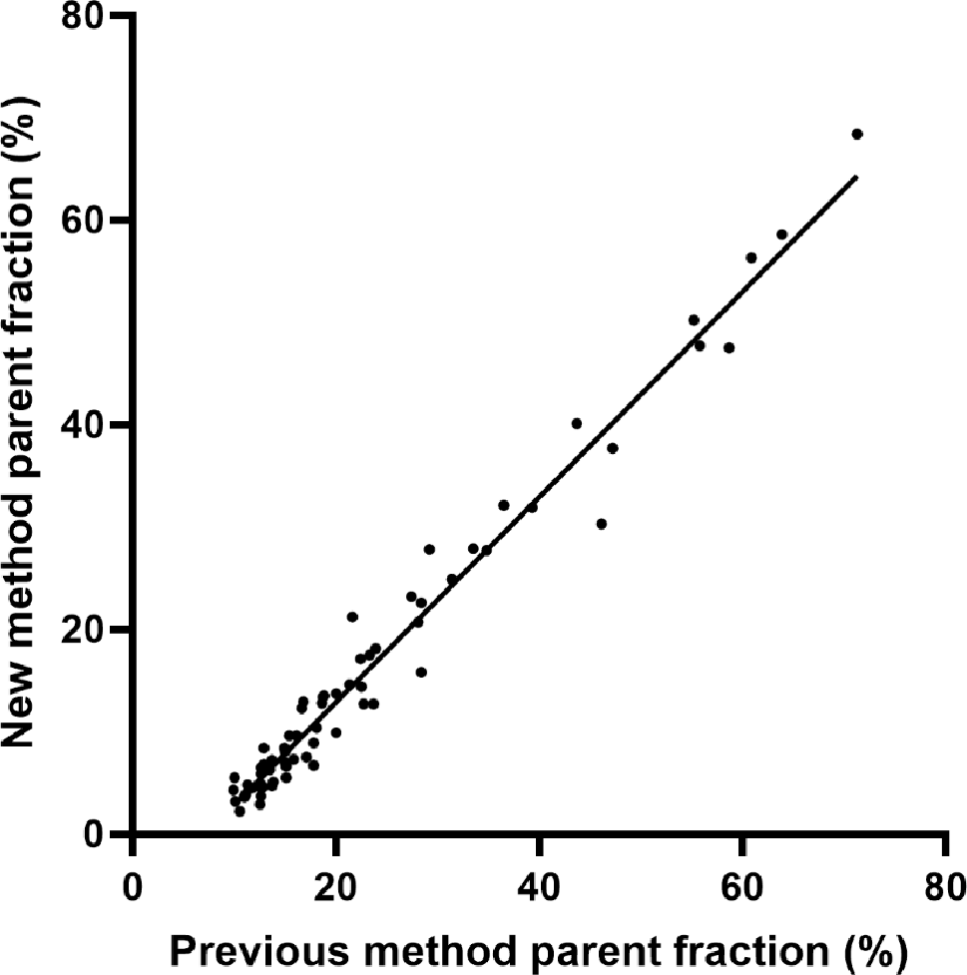
Correlation of parent fraction results (%) between the two tested methods. Parent fraction values are plotted from all the subjects analyzed with both the previous and new methods (*n* = 5 for fasted visits; *n* = 5 for postprandial visits, data collected from four different subjects)

The parent fraction values correlated well between the used methods (goodness of linear fit, R^2^ = 0.99 for fasted, 0.98 for postprandial), showing that the previous method consistently overestimated the parent fraction over the study time-points (Fig. 4). We found a difference of 7 percentage points between the methods, which was also approximately the difference of the metabolites migrating above the further travelling peak of [^18^F]FTHA (Fig. 1 and Supplemental Fig. 1).Therefore, deducting 7.2 percentage points (the intercept of the correlation line in Fig. 4) from all time-points analyzed using the previous method provided an accurate correction to compensate for the difference between the two methods (Supplemental Fig. 2). The samples analyzed only using the previous method were corrected using this correction factor, enabling comparable analysis of the differences between fasted and postprandial measurements, and test-retest results.

We compared the parent fraction curves and the corresponding AUCs of the same individuals at repeated study visits. Compared to the fasted state, in the postprandial state, all subjects had a faster [^18^F]FTHA metabolism and the parent fraction curve reached a plateau stage earlier (Fig. 2). On average, the mean AUC (0–60 min) was 42% larger (*p* < 0.0001) in the fasted state (mean AUC of visit 1 and 2 = 2135; *n* = 34) than in the postprandial state (mean AUC of visit 3 and 4 = 1502; *n* = 32) (Supplemental Table 3).

The intra-individual test-retest visits yielded repeatable parent fraction results in consecutive visits. Figure 2C presents an example of the parent fraction curves of a study subject. On average, we observed a 6.1 ± 4.3% difference in the parent fraction AUCs (0–60 min) between the two fasted visits (both test and re-test visits of 17 subjects), and a 7.9 ± 5.4% difference between the two postprandial visits (both test and re-test visits of 15 subjects) (Supplemental Table 3). The *p* = 0.31 for fasted and 0.74 for postprandial groups strongly indicated non-significant difference in the identical retest visit as hypothesized.

### Plasma TAC

**Fig. 5:**
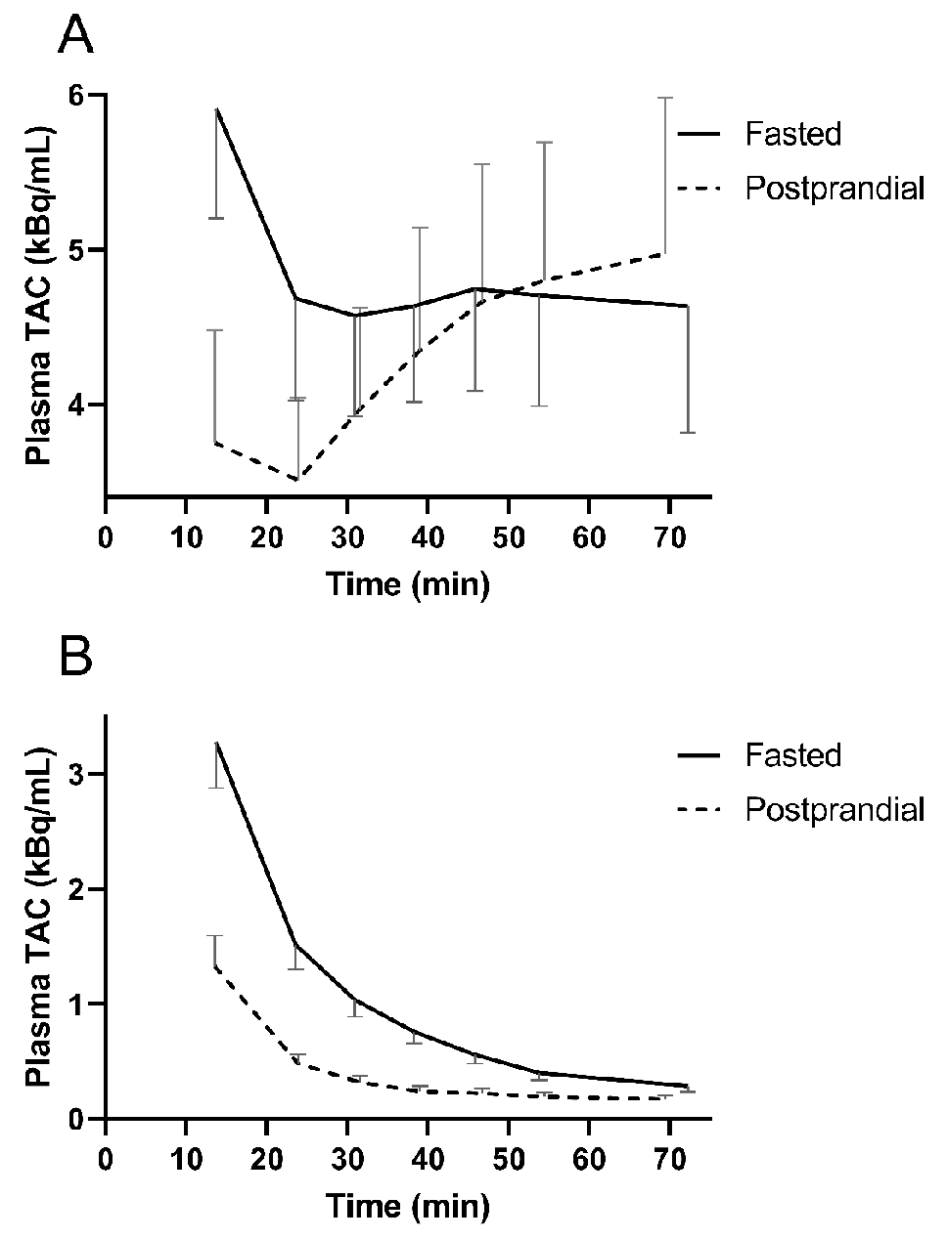
(**A**) Plasma time-activity curves (TACs) from collected blood samples during fasted visits (continuous line) and postprandial visits (dashed line). (**B**) Parent fraction-corrected TACs using the new radiometabolite analysis (RMA) method. Data presented are mean and SD. *n* = 34 for fasted, and *n* = 34 for postprandial visits

Figure 5 shows the mean plasma TAC, and the corresponding radiometabolite-corrected TAC, from the first manual blood sample time-point onwards (approximately 14 min). The initial plasma TAC was markedly higher at the fasting visit than in the postprandial protocol. However, after about 50 min, the mean postprandial TAC superseded the mean fasted TAC. During postprandial visits, the total radioactivity concentration markedly increased after around 24 min, showing a significant mean increase of 42 ± 20% from the lowest point within the first 70 min (*p* < 0.0001, *n* = 34 postprandial visits). In contrast, during fasting visits, the plasma TAC reached a plateau after 24 min. With both protocols, the plasma radiometabolite-corrected [^18^F]FTHA concentration followed descending curves.

We determined the plasma SUV repeatability by interpolating the plasma SUV values to a fixed time range (14–60 min) for all subjects, and calculating the percentage of the mean absolute difference of the generated AUCs from the consecutive visits (Supplemental Table 3). The mean absolute difference between individual subjects’ test-retest plasma SUV AUCs was 8.1 ± 4.4% (*p* = 0.29) for the fasted visits (*n* = 17), and 9.1 ± 6.5% (*p* = 0.67) for postprandial visits (*n* = 16), indicating good repeatability as neither retest was significantly different. The mean SUV AUC difference was 2.5% for the fasted protocol (visit 1 = 82.1 and visit 2 = 84.1), and 1.0% for the postprandial protocol (visit 3 = 73.3 and visit 4 = 72.8).

## Discussion

In this study, we introduced a new improved radio-TLC RMA method for [^18^F]FTHA, and demonstrated its value for clinical use. Compared to the previously used method, our method separated more radiometabolites from each other, and separated one or more radiometabolites that previously co-eluted with [^18^F]FTHA. Consequently, the parent fraction values obtained with the new method reached lower levels over time, revealing that [^18^F]FTHA metabolism was faster and more complete than was indicated by the previous method. Furthermore, we conducted RMA using both methods using aliquots of the same samples from a subset of subjects, enabling us to calculate a parent fraction correction factor for subjects analyzed using either method in this study. We found a robust correction factor for the previously used method, which was then used to correct all the sample time-points (Supplemental Fig. 2) allowing further evaluations of larger subject groups.

We observed that some radiometabolites formed rapidly, but their fraction in plasma declined over time via further metabolism into other radiometabolites and excretion from plasma (Figs. 1 and 3). The ability to separate more radiometabolites from each other could be useful because it can enable the modeling of multiple radiometabolite compartments [21]. However, separating more radiometabolites from each other is only a secondary objective in RMA.

To achieve sufficient R_s_, the new method required a 20-minute increase of elution time compared to the previous method. However, due to the 109.8-minute half-life of fluorine-18, this increased elution time caused only a 12% reduction in signal intensity; therefore, the peak intensities remained well above the lowest quantification levels. The optimal ratio of plasma protein to precipitation solvent was 1:1.4 (plasma: methanol + 0.4% acetic acid). Chromatographic separation was not notably affected when using different ratios within the range of 1:1–1:1.8, but the radioactivity concentration of the supernatant was highest when using a ratio of 1:1.4. This may have been because the 1:1.4 ratio has a sufficiently high organic composition to precipitate the plasma proteins, disrupt the binding of radioactive components to plasma components, and dissolve the components into the supernatant phase rather than the protein pellet. Dilution with increased precipitation solvent may counterbalance the slight increase of the extraction efficiency. In general, the optimal ratio should minimize dissolved plasma protein remnants that might interfere with chromatography, while simultaneously maximizing the radioactivity concentration [22].

One new observation from our present study is that the metabolism rate of each study subject was consistent on the retest day, with low deviation of the parent fraction curves between two standardized visits (Fig. 2C). The percentage of the mean difference in the parent fraction AUC between the standardized test-retest visits reflects the robustness of the radio-TLC method, as well as the consistency of an individual subject’s [^18^F]FTHA metabolism, and clearance rates of radioactive components out of plasma. Based on the steadiness of the results between identical visits, future studies should consider whether fewer blood samples are actually needed in similar study subject cohorts, or whether the parent fraction curves could be modeled based on previous results, by correcting the curve with the few checkpoint samples. Under identical retest settings, blood sampling could be even omitted through the use of previous parent fraction data.

Interestingly, the non-radiometabolite-corrected postprandial state plasma TAC revealed a return of ^18^F-radioactivity to plasma after the 24-min time-point sample (Fig. 5), which is similar to previously reported findings during hyperinsulinemia [10]. However, with accurate parent fraction correction, the metabolite-corrected plasma TAC showed that the source was not [^18^F]FTHA, but rather the returning radiometabolites (Fig. 5). This phenomenon was likely due to the rapid metabolism of [^18^F]FTHA into other lipids in the liver [10], and their consequential efflux back to the plasma. This indicates that postprandially (approximately 2 h after a meal), the ^18^F-radioactivity is not exclusively trapped inside cells in all tissues [3], because the [^18^F]FTHA radiometabolites start to partly re-circulate back into plasma [10, 23]. During fasting, the total radioactivity in plasma did not similarly increase during the one-hour sample collection time, indicating that this phenomenon was much slower in the fasted state.

Our results indicate that when using the previous method, the presence of co-eluting radiometabolites with one of the [^18^F]FTHA bands caused the parent fraction curve to remain above 10% at the plateau region, rather than this indicating that the [^18^F]FTHA plasma efflux and influx rates had reached equilibrium. Previous studies have also indicated irreversible uptake of [^18^F]FTHA [10, 24]. The parent compound band, which did not diminish to zero according to the previous method, revealed that the co-eluting radiometabolite reached an equilibrium with the uptake and metabolism rates of [^18^F]FTHA, as its fraction of the total radioactivity in plasma remained relatively constant, as determined by subtracting the parent fraction curve obtained using the new method from that obtained with the previous method (Fig. 2). The impact of the difference between the two methods can be estimated, and it seems likely that this difference has not had a large effect on group comparisons in previous studies, since both groups exhibited the same magnitude of error when using the same method. However, there has likely been a greater effect on individual value quantification, especially in studies covering the later time-points, which exhibit the largest fraction of the unseparated radiometabolite.

This study was conducted only with participants with overweight or obesity and therefore direct conclusions cannot be drawn to other demographic groups. It is possible that the lipid handling of lean or normal weight subjects could have different kinetics, which remains to be studied further [25].

Only a few [^18^F]FTHA parent fraction curves (analyzed up to 30–50 min) from human plasma samples have been previously published [16, 17, 26]. These curves mostly show that the parent fraction seems to plateau at 20–30%, and that the SD is much larger than that determined with our new method [16, 26]. The inability to accurately analyze the radiometabolites, especially towards the end of the dynamic [^18^F]FTHA acquisition, has a clear impact on Patlak analysis, which uses the linear region of the curve after the distribution phase. The effect can be large especially in the postprandial setting, where we observed a rapid decline in the parent fraction, reaching nearly zero after approximately 30 min. Correct evaluation of the parent fraction is vital for accurate kinetic calculations.

## Conclusions

Here we introduced a newly developed radio-TLC method for [^18^F]FTHA RMA, which enhances the separation of [^18^F]FTHA from its radiometabolites, compared to previous methods. We conducted a test-retest study under both fasted and postprandial conditions, which demonstrated consistent repeatability, ensuring reliable acquisition of quantitative data for modeling [^18^F]FTHA PET data. Our use of an accurate RMA method revealed clear differences in the metabolic rates across different study settings, and facilitated precise parent fraction correction.

### Electronic supplementary material

Below is the link to the electronic supplementary material.

Supplementary Material 1

## Data Availability

The analysed datasets are available from the corresponding author on reasonable request.

## Abbreviations

[^18^F]FTHA 14-(*R,S*)-: [^18^F]fluoro‐6‐thia‐heptadecanoic acid
AUC: Area under the curve
FFA: Free fatty acid
K_i_: Net influx rate
NEFA: Non-esterified fatty acid
PET: Positron emission tomography
Radio-TLC: Thin-layer chromatography combined with digital autoradiography
RMA: Radiometabolite analysis
R_s_: Resolution
SD: Standard deviation
SUV: Standardized uptake value
TAC: Time-activity curve
TLC: Thin-layer chromatography
