# Supplementary material for: New improved radiometabolite analysis method for [18F]FTHA from human plasma: a test-retest study with postprandial and fasting state
Source: EJNMMI Res. 2024 Jun 13;14:53. doi: 10.1186/s13550-024-01114-5 (PMC11176130; doi:10.1186/s13550-024-01114-5)
Supplement: Supplementary file 1 — Supplementary Material 1 [file 13550_2024_1114_MOESM1_ESM.docx]

**Supplemental figure 1:**


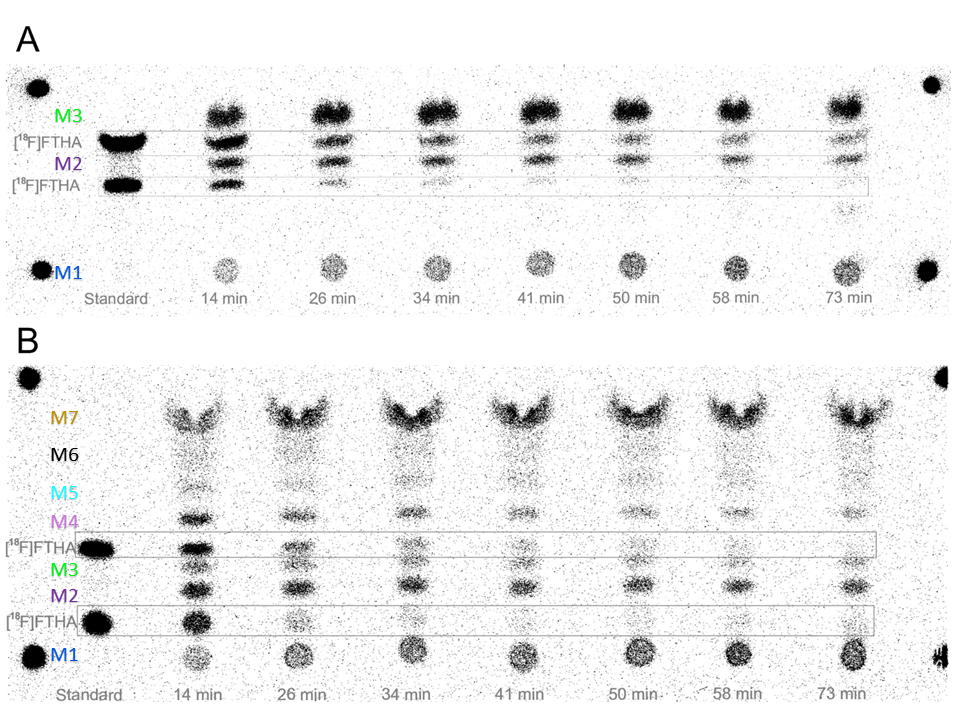


**SUPPLEMENTAL Fig. 1.** Representative autoradiographs showing separation of the parent fraction of [^18^F]FTHA into two separate bands, along with its radioactive metabolites, on a HPTLC plate. Separations were performed on the same plasma samples using both the previous method (A) and the new method (B). Corner spots indicate the application and solvent front line.**Supplemental figure 2:**

**SUPPLEMENTAL Fig. 2.** Percentages of parent [^18^F]FTHA out of all radioactivity in the sample, analyzed with the previous method (blue), the new method (dashed red), and the correction factor-corrected previous method (black). Data presented are mean and SD. *n* = 10 visits for the previous method, and *n* = 10 visits for the new method. Plasma samples from all subjects were analyzed with both methods, side-by-side.


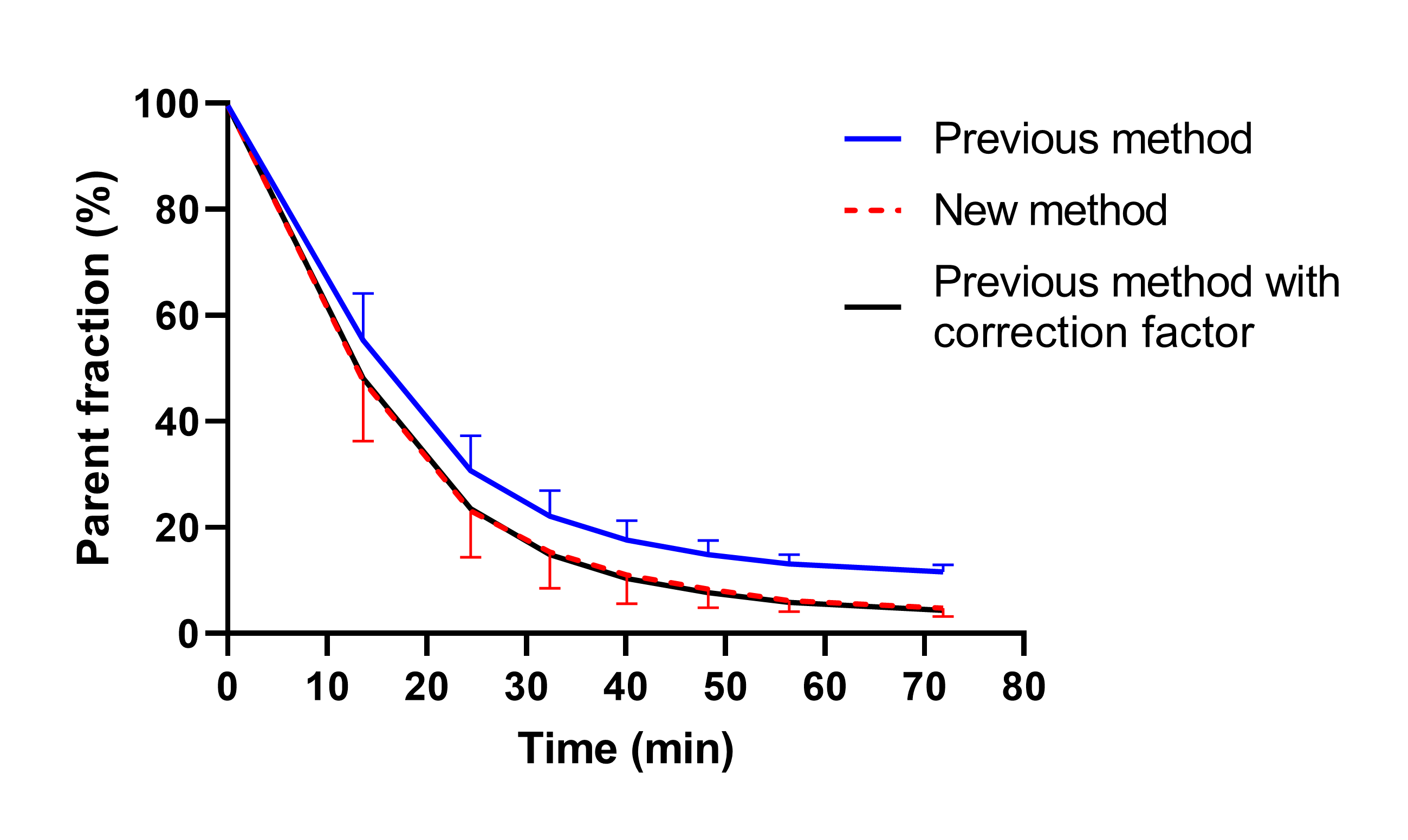


| **SUPPLEMENTAL TABLE 1**  Sample Preparation Methods, TLC Plates, and Eluents Tested During Development of a New Chromatographic Method for [^18^F]FTHA Radiometabolite Analysis | | | | |
| --- | --- | --- | --- | --- |
| Precipitation solvents | Plasma to precipitation solvent ratios | TLC plates | TLC Plate Reference numbers | Eluents |
| MeOH + 0.4% AcOH,  MeOH,  ACN + 0.4% AcOH,  ACN | 1:1,  1:1.4,  1:1.8 | Merck HPTLC RP18 old LOT, manufactured prior to 12/20 (glass, used in the previous method) | 1.05914.0001 | 68–95% MeOH, 0–10% ACN, 5–32% H_2_O and 0.13% AcOH |
|  |  | Merck HPTLC RP18 new LOT, manufactured 12/20 or after (glass, chosen for the new method) | 1.05914.0001 |  |
|  |  | Merck HPTLC RP18 (aluminium backed) | 1.13724.0001 |  |
|  |  | Merck TLC RP18 | 1.05559.0001 |  |
|  |  | Macherey-Nagel HPTLC/TLC precoated plates Nano-SIL C18-100 | 811052 |  |
|  |  | Macherey-Nagel HPTLC/TLC precoated plates Nano-SIL C18-50 | 811054 |  |
|  |  | Merck TLC silica gel 60 | 1.05554.0001 | 5:1:0,1 DCM:MeOH:TFA,  9:1:0,01 DMC:MeOH:AcOH |
| MeOH = methanol, ACN = acetonitrile, DCM = dichloromethane, TFA = trifluoroacetic acid, AcOH = acetic  acid. | | | | |

**SUPPLEMENTAL TABLE 2**

Areas Under the Curve of Parent Fractions from Plasma Samples Analyzed Using the Previous and New Methods, and the Percentage of Absolute Difference for Fasted and Postprandial Visits (0–60 min)

|  |  | Previous Method | new Method | Difference (%) |
| --- | --- | --- | --- | --- |
| Parent fraction AUC FASTED | Mean | 2479 | 2142 | 16.2 |
|  | SD | 156 | 212 | 5.5 |
|  | n (visits) | 5 | 5 |  |
| Parent fraction AUC PP | Mean | 2038 | 1490 | 37.2 |
|  | SD | 107 | 126 | 7.0 |
|  | n (visits) | 5 | 5 |  |

AUC = Area Under the Curve

PP = Postprandial

## The mean absolute difference (%) was calculated as the mean of absolute differences of the AUCs of each individual’s parent fraction results from the previous and new method, divided by that subject’s new method AUC result. Data are presented as mean and standard deviation (SD).SUPPLEMENTAL TABLE 3

Mean Area Under the Curve Values of the Parent Fraction Curves (0–60 min) and Plasma SUV Curves (14–60 min) from Different Visits

|  |  | Visit 1 | Visit 2 | Mean Difference (%) |
| --- | --- | --- | --- | --- |
| Parent fraction AUC FASTED | Mean | 2178 | 2092 | 6.1 |
|  | SD | 236 | 232 | 4.3 |
|  | n | 17 | 17 |  |
|  |  | **VISIT 3** | **VISIT 4** |  |
| Parent fraction AUC PP | Mean | 1507 | 1496 | 7.9 |
|  | SD | 226 | 281 | 5.4 |
|  | n | 17 | 15 |  |
|  |  | **VISIT 1** | **VISIT 2** |  |
| Plasma SUV AUC FASTED | Mean | 82.1 | 84.1 | 8.1 |
|  | SD | 9.1 | 7.8 | 4.4 |
|  | n | 17 | 17 |  |
|  |  | **VISIT 3** | **VISIT 4** |  |
| plasma SUV AUC PP | Mean | 73.3 | 72.8 | 9.1 |
|  | SD | 11.8 | 9.0 | 6.5 |
|  | n | 17 | 16 |  |

AUC = Area Under the Curve

PP = Postprandial

Data are presented as mean and standard deviation (SD).

Mean difference (%) = the mean of the difference between the AUC values of the consecutive visits, analyzed as the percentage of the absolute difference of the consecutive visits of each subject, divided by the mean AUC of the subjects’ consecutive visit, for fasted and postprandial (PP) visits separately.

## Residual [^18^F]FTHA in Injection Tubing

As the [^18^F]FTHA injection was performed while the study subject was inside the PET/MRI scanner, an unusually long (1.5-m) tubing was used to intravenously inject the tracer. Since [^18^F]FTHA is a fatty acid analog with known interactions with plastics, we optimized the injection syringe flushing process. After the injection, we performed a rapid saline flush (3 mL) from the same injection syringe, and additional flushes (2 × 10 mL) from a 10-mL syringe, using an upstream three-way valve (Discofix® C 3-way Stopcock Connection Tubing). This protocol was designed to maintain a bolus injection. We used a low-sorbing and flexible tubing material (BD: 3 × 50 cm, Extension set, PB-G40705; Becton, Dickinson and Company, Franklin Lakes, NJ; total internal volume of 1.2 mL), to minimize the retention of radioactivity. The tubing was removed from the cannula after the first dynamic imaging acquisition session (10 min), and measured with the injection syringe in an isotope calibrator. The residual radioactivity was decay-corrected to the time of injection, and the residual activity within the tubing and syringe was subtracted from the initial amount in the syringe at the time of injection. This calculation enabled us to determine the true injected radioactivity, which is crucial for accurate standardized uptake value (SUV) analysis.
